# Bile acid nephropathy induced by anabolic steroids: A case report and review of the literature 

**DOI:** 10.5414/CNCS110711

**Published:** 2021-11-09

**Authors:** Hamdan Al Awadhi, Sarah Al Qassimi, Aya Akhras, Leal Herlitz, Muriel Ghosn

**Affiliations:** 1 Division of Internal Medicine, Department of Medicine, Cleveland Clinic Abu Dhabi Hospital, Abu Dhabi, UAE,; 2 Department of Pathology, Cleveland Clinic, Cleveland, Ohio, USA, and; 3 Division of Nephrology, Department of Medicine, Cleveland Clinic Abu Dhabi Hospital, Abu Dhabi, UAE; * Both authors contributed equally to this work.

**Keywords:** cholemic nephropathy, bile acid nephropathy, drug-induced liver injury, anabolic steroids

## Abstract

Bile acid nephropathy also known as cholemic nephropathy is a rare and overlooked form of acute kidney injury that occurs in the setting of severe hyperbilirubinemia. The exact etiology remains unknown, and there is a lack of treatment guidelines for this clinical condition. Anabolic steroids are known to cause hepatoxicity occasionally leading to acute kidney injury. We report the case of a 27-year-old male patient who developed bile acid nephropathy as a result of severe hyperbilirubinemia secondary to anabolic steroids-induced liver injury. He was conservatively managed. We review the current literature touching on the etiology, pathophysiology, diagnosis, and management of bile acid nephropathy in an attempt to shed light on this clinical condition, which may present as a diagnostic and therapeutic challenge.

## Introduction 

Bile acid nephropathy is a form of acute kidney injury seen in patients with severe direct hyperbilirubinemia. Its incidence is poorly reported, but the risk of developing this condition increases with the duration and severity of hyperbilirubinemia, usually with a total bilirubin level above 20 mg/dL [1]. Suggested etiologies reported in the literature include conditions that can lead to significant hyperbilirubinemia such as alcoholic cirrhosis [1, 2], hepatitis C [3], drug-induced liver injury including injury secondary to anabolic steroids [8, 9, 10, 11, 12, 13], pancreatic tumors, common bile duct stones [5], and infectious mononucleosis [7]. Kidney biopsy is considered the gold standard method for diagnosis. 

## Case report 

A 27-year-old male patient with no significant past medical history reported using anabolic steroids for almost 1 month to augment his gym performance. He was taking the following supplements: drostanolone, testosterone, and stanozol. Three months later, he endorsed symptoms of fatigue, diffuse abdominal pain with yellow tinge to his conjunctivae as well as pale stools and dark urine. He visited a local facility where he was found to have cholestatic liver injury. Family history was negative for kidney or liver diseases. Social history was negative for alcohol or tobacco use, intravenous drug abuse, tattoos, history of blood transfusions or high-risk sexual behavior. Medication history included the use of levocetirizine for allergies but no other chronic medication use. The liver injury was attributed to the anabolic steroids he took. He was treated with oral steroids and subsequently developed acute kidney injury requiring initiation of dialysis. 

One month later, he was transferred to our facility for continuity of management. On physical examination, he was alert and oriented to time, place, and person. There was evidence of diffuse jaundice and scleral icterus. Abdomen was soft with tenderness noted in the epigastric region. There were multiple bleeding skin excoriations noted. Other system examinations were normal. Initial laboratory test results are summarized in Table 1. 

He was diagnosed with anabolic steroid-induced liver injury and was slowly tapered off steroids (they were stopped after a 2-week course).

**Figure 1:**
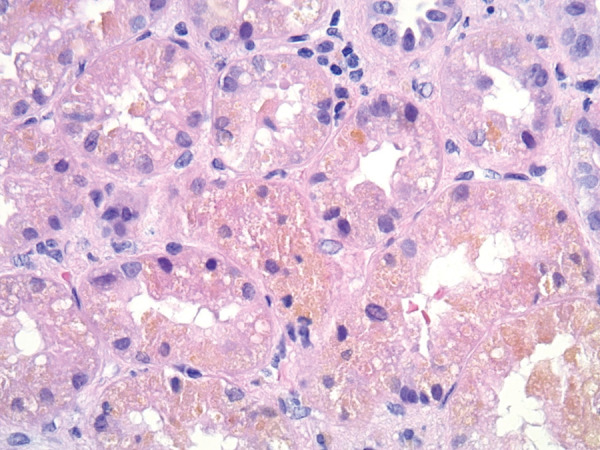
H & E (× 400) shows accumulated brown pigment in proximal tubular cytoplasm consistent with bile.

**Figure 2:**
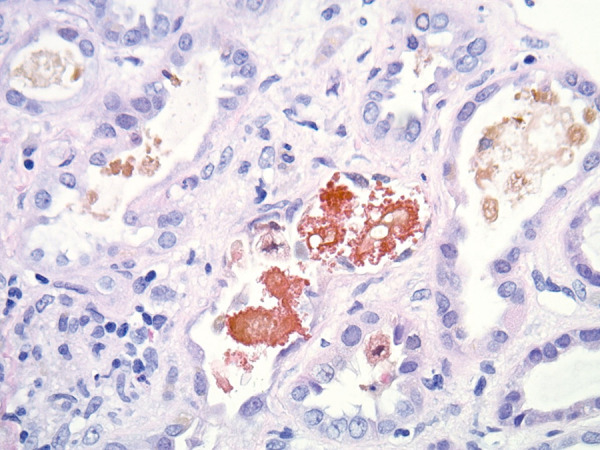
H & E (× 400) shows a distal tubule with a bile cast.

His bilirubin levels remained static. His kidney function initially improved, and dialysis was stopped. However, 2 weeks into his admission, his kidney function started to gradually worsen again. At this stage, a kidney biopsy was performed and showed diffuse acute tubular injury and the presence of intracellular pigmented material and multifocal casts. Hall’s staining for bile highlighted the bile casts. There was minimal tubular atrophy and interstitial fibrosis (Figure 1, Figure 2) 

He was eventually restarted on intermittent hemodialysis. His bilirubin levels remained elevated throughout his hospital stay but with normal liver function tests otherwise. He was managed with ursodeoxycholic acid and was off steroids as mentioned above. 

Plasmapheresis was considered, however, the patient sustained a significant perinephric hematoma following the kidney biopsy as well as a spontaneous left arm hematoma thus it was deferred. 

**Figure 3:**
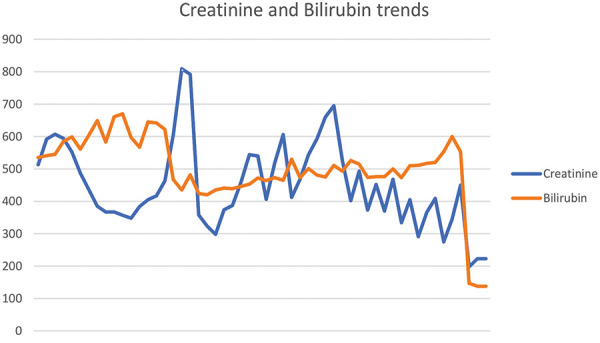
Creatinine and bilirubin trends.

He was discharged home on intermittent hemodialysis. His kidney function slowly improved, and hemodialysis was eventually stopped 2 months later. Serum creatinine was 2.6 mg/dL (201 µmole/L) at his last follow-up. Total serum bilirubin was 4.6 mg/dL (79 µmol/L). Table 1 summarizes the results of the laboratory tests at his last follow-up. Figure 3 shows creatinine and bilirubin trends throughout hospital admission. 

## Discussion 

We report a case of bile acid nephropathy secondary to severe hyperbilirubinemia related to steroid-induced liver injury in a 27-year-old male patient who was managed with hemodialysis. A brief overview of the etiology, pathophysiology, and management of this condition will be presented. 

### Prevalence, etiology, and pathophysiology 

Bile acid nephropathy is a form of acute kidney injury occurring in patients with severe direct hyperbilirubinemia. Quincke first described it in 1899 after reviewing autopsies of patients with jaundice and acute kidney insufficiency. In 1922, Hessler et al. showed that severe jaundice was associated with the presence of free bilirubin in the urine of dogs and humans. Subsequently, in 1937, Elsom et al.observed that jaundice could cause kidney impairment that is reversible following the improvement of bilirubin levels [32, 33]. While this entity was first recognized in the late 1800s and early 1900s, it has been largely overlooked and underreported in the recent years for various reasons: decreased awareness of this condition by the clinicians, avoidance of kidney biopsies in patients with liver diseases due to the increased bleeding risk, difficulty identifying the bile casts on routine H & E sections and the uncertainty of their clinical significance [2, 33]. 

Many recent clinicopathologic and post-mortem kidney biopsy studies show that bile acid nephropathy is more frequent then previously sought and should be included in the differential diagnosis of acute kidney injury in patients with liver diseases. Van Slambrouck et al. [1] conducted a clinicopathologic study of 44 subjects (41 autopsies and 3 kidney biopsies) from jaundiced patients at the University of Chicago and documented the presence of bile casts in 55% of the cases, including 85% of those with a pre-mortem diagnosis of hepatorenal syndrome. In 94 autopsy cases of liver cirrhosis, bile casts were demonstrated in 55% of them by Foshat et al. [3]. Hepatitis C virus infection accounted for 52% of the cases of cirrhosis. Nayak et al. [4] conducted a post-mortem kidney biopsy study on 127 patients admitted with acute on chronic liver failure and decompensated liver cirrhosis with a clinical diagnosis of acute kidney injury secondary to hepatorenal syndrome. Bile acid nephropathy was detected in 44.8% of all the post-mortem kidney biopsy specimens and in 72.1% of the patients with acute liver failure superimposed on chronic liver failure. 

These studies not only show the relatively high prevalence of bile acid nephropathy in patients with liver disorders but also suggest that this entity could contribute to the delayed or poor treatment response seen in patients with acute kidney injury presumed secondary to hepatorenal syndrome. 

Bile acid nephropathy has been described in patients with obstructive cholestasis secondary to stones in the common bile duct [5], acute alcoholic hepatitis [2], alcoholic liver cirrhosis [1], liver cirrhosis secondary to chronic hepatitis C and chronic hepatitis B [3], status post wedge resection of liver in a patient with colorectal cancer [6], in the setting of infection with mononucleosis [7], and following the use of anabolic steroids similarly to our patient [8, 9, 10, 11, 12, 13]. Any hepatic or extrahepatic insult leading to hyperbilirubinemia may result in this condition, particularly with bilirubin levels exceeding 20 mg/dL. 

The pathophysiology is poorly understood but is thought to involve multiple mechanisms, which include uncoupling of mitochondrial phosphorylation and decrease in ATPase activity leading to direct tubular epithelial injury from oxidative damage, disturbances of renal hemodynamic responses, and formation of casts [2, 11, 32]. This is particularly true with bilirubin levels exceeding 20 mg/dL although lower levels were implicated in the study by Nayak et al. [4]. 

### Histopathology 

The pathology induced by hyperbilirubinemia is mainly limited to the tubules. Many case reports of bile acid nephropathy have demonstrated bilirubin cast deposition predominantly in the distal renal tubules and minimally in the proximal tubules. The bile casts can be identified by the Hall stain, which detects bilirubin. This stain utilizes Fouchet reagent, which converts bilirubin to biliverdin, thus yielding a green color. 

Acute tubular necrosis is also a prominent feature and manifests itself by the loss of tubular brush borders, attenuated cytoplasm of tubular cells, dilatation of the tubular lumen, cytoplasmic vacuolization, apical blebbing, and tubular necrosis and desquamation [1, 2, 3]. 

Immunofluorescence and electron microscopy are usually unremarkable, although, ultrastructurally in some cases the bile casts can appear filamentous with a focal whorled pattern and moderately to highly electron-dense within distal tubular lumina. Electron microscopy can also show bile particles in the cytoplasm of proximal or distal tubular cells as well as enlargement, disarray, and cristae disorganization of mitochondriae [11]. 

### Management 

Owing to the rarity of this condition and the lack of robust randomized clinical trials, there are currently no accepted treatment guidelines. Rather, the therapeutic approach usually focuses on decreasing bilirubin levels and reversing the cause of the liver injury. The earlier the interventions, the better the outcomes. It is important to recognize that the kidney damage might become irreversible later in the disease course, requiring kidney and possibly liver transplantation. Reduction in bilirubin levels can be achieved via endoscopic retrograde cholangiopancreatography with or without stenting to relieve the obstruction in cases of biliary stones or tumors [5]. In the absence of obstruction, excess bilirubin and bile salts can be removed via extracorporeal therapies such as plasmapheresis and albumin dialysis. However, these therapies are not supported by randomized controlled trials, and their use is based on case reports or case series. 

Plasmapheresis was shown to effectively reduce bilirubin levels in a small study published by Keklik et al. [14] that included patients with severe hyperbilirubinemia secondary to liver cirrhosis, fulminant hepatitis, and liver cancer. Two cases of anabolic steroid-induced bile acid nephropathy successfully managed with plasmapheresis without the need for renal replacement therapy were reported by El Khoury et al. [9] and by Flores et al. [15]. Ocon et al. [16] also reported a case of thyrotoxicosis-induced bile acid nephropathy successfully managed with plasmapheresis and temporary hemodialysis followed by full renal recovery. 

Albumin dialysis is based on the removal of unwanted albumin-bound and water-soluble substances such as bilirubin, bile acids, and other hepatotoxins that are mostly albumin bound. It can be done using two modalities: molecular absorbent recirculating system (MARS) and single-pass albumin dialysis (SPAD) [17]. 

While a few small studies showed that MARS improved portal pressure [18, 19], hyperdynamic circulation [20], hepatic encephalopathy [21, 22], and hepatorenal syndrome [23] in patients with acute liver failure, a mortality benefit was not demonstrated [24, 25, 26]. SPAD was compared to MARS in terms of reduction of bilirubin levels and influence on kidney parameters such as urea and creatinine. Both systems reduced bilirubin levels similarly, but MARS decreased serum creatinine and bile acid levels more significantly [27] 

Few papers looked at the use of albumin dialysis specifically for the treatment of bile acid nephropathy; Saich et al. [28] described the successful use of MARS in a patient with acute kidney injury secondary to benign recurrent intrahepatic cholestasis that failed medical therapy. Sens et al. [29] reported a drastic improvement of the kidney function in a patient with bile acid nephropathy following 1 session of MARS and 8 sessions of SPAD. Similarly, Castellanos et al. [13] treated a patient with bile acid nephropathy secondary to anabolic steroid-induced liver injury with hemodialysis followed by MARS and continuous venovenous hemodialysis (CVVHD). This was followed by a marked improvement in kidney function and withdrawal of dialysis. A few centers also reported their experience with SPAD, which allowed effective reduction of bilirubin levels in patients with acute liver failure and severe hyperbilirubinemia with or without acute kidney failure [30, 31]. 

### Outcomes 

In the literature, there are a handful of reported cases of bile acid nephropathy secondary to anabolic steroid-induced liver injury. The patients presented with severe hyperbilirubinemia and acute kidney injury an average of 1 – 6 months following anabolic steroid use. Approximately 25% of them had a slow spontaneous recovery without the need for dialysis or other supportive treatments. The average time for recovery was 3 – 5 months [8, 11]. 40% of them required temporary dialysis followed by partial or complete kidney recovery [10, 12]. Plasmapheresis was used in 25% of the cases and led to a complete recovery without the need for dialysis [9, 15], and finally MARS with CVVHD were done in 10% of the patients, with normalization of kidney function and withdrawal of CVVHD [13]. 

## Conclusion 

Our case highlights bile acid nephropathy as an entity that remains under-diagnosed. It is important to consider this condition in the differential diagnosis of acute kidney injury associated with cholestatic liver disease. While some conditions, such as cholestasis secondary to bile duct stones, are rapidly reversible, others, including anabolic steroid-induced cholestatic liver injury, can lead to a profound kidney injury that might require renal replacement therapy with significantly delayed recovery. This highlights the need for an early diagnosis and management before irreversible kidney injury ensues. Kidney biopsy remains the gold standard method for diagnosis but is still rarely performed due to the higher risk of bleeding in this patient population. Treatment options are limited and are largely supportive. There are reports of the successful use of plasmapheresis, MARS, or SPAD in treating bile acid nephropathy but larger randomized controlled studies are required before recommending the universal use of such therapies. 

## Funding 

None. 

## Conflict of interest 

The authors declare that they have no relevant financial interest. 

**Table 1:** Laboratory values on admission and at last follow-up.

Laboratory tests	Normal range	On admission	At last follow-up
White blood cells	4,500 – 11,000 µ/L (4.5 – 11 × 10^9^/L)	10,900 µ/L (10.9 × 10^9^/L)	9,100 µ/L (9.1 × 10^9^/L)
Hemoglobin	13.2 – 17.3 g/dL (132 – 173 g/L)	7.9 g/dL (79 g/L)	13.3 g/dL (133 g/L)
Platelets	140 – 400 µL (140 – 400 × 10^9^/L)	255 × 10^3^/ µL (255 × 10^9^/L)	392 × 10^3^/ µL (392 × 10^9^/L)
Sodium	136 – 145 mEq/L (136 – 145 mmol/L)	138 mEq/L (138 mmol/L)	141 mEq/L (141 mmol/L)
Potassium	3.6 – 4.8 mEq/L (3.6 – 4.8 mmol/L)	4 mEq/L (4 mmol/L)	4.2 mEq/L (4 mmol/L)
Chloride	98 – 107 mEq/L (98 – 107 mmol/L)	98 mEq/L (98 mmol/L)	106 mEq/L (106 mmol/L)
CO_2_	22 – 29 mEq/L (22 – 29 mmol/L)	22 mEq/L (22 mmol/L)	17 mEq/L (17 mmol/L)
Urea	7.8 – 22.7 mg/dL (2.8 – 8.1 mmol/L)	54.9 mg/dL (19.6 mmol/L)	36.7 mg/dL (13.1 mmol/L)
Calcium	8.6 – 10.2 mg/dL (2.15 – 2.55 mmol/L)	9.28 mg/dL (2.32 mmol/L)	10.16 mg/dL (2.54 mmol/L)
Alanine aminotransferase (ALT)	17 – 63 U/L	61 U/L	61 U/L
Aspartate aminotransferase (AST)	0 – 39 U/L	74 U/L	74 U/L
Alkaline phosphatase	40 – 129 U/L	203 U/L	203 U/L
Bilirubin, total	0.3 – 1.23 mg/dL (5 – 21 µmol/L)	31.27 mg/dL (535 µmol/L)	4.6 mg/dL (79 µmol/L)
Creatinine	0.67 – 1.18 mg/dL (59 – 104 µmol/L)	6.7 mg/dL (513 µmol/L)	2.6 mg/dL (201 µmol/L)
Protein, total	6.6 – 8.7 g/dL (66 – 87 g/L)	5.2 g/dL (52 g/L)	7.5 g/dL (75 g/L)
International normalized ratio (INR)		1.3	
Ammonia	15 – 45 μg/dL (11 to 32 μmol/L)	17 μg/dL (10 μmol/L)
